# MedSlice: fine-tuned large language models for secure clinical note sectioning

**DOI:** 10.1093/jamiaopen/ooaf179

**Published:** 2026-01-13

**Authors:** Joshua Davis, Thomas Sounack, Kate Sciacca, Jessie M Brain, Brigitte N Durieux, Nicole D Agaronnik, Charlotta Lindvall

**Affiliations:** Department of Supportive Oncology, Dana-Farber Cancer Institute, Boston, MA 02215, United States; Albany Medical College, Albany, NY 12208, United States; Department of Supportive Oncology, Dana-Farber Cancer Institute, Boston, MA 02215, United States; Department of Supportive Oncology, Dana-Farber Cancer Institute, Boston, MA 02215, United States; Brigham and Women’s Hospital, Boston, MA 02115, United States; Department of Supportive Oncology, Dana-Farber Cancer Institute, Boston, MA 02215, United States; Brigham and Women’s Hospital, Boston, MA 02115, United States; Department of Supportive Oncology, Dana-Farber Cancer Institute, Boston, MA 02215, United States; Department of Family Medicine, McGill University, Montreal, QC H3S 1Z1, Canada; Department of Supportive Oncology, Dana-Farber Cancer Institute, Boston, MA 02215, United States; Harvard Medical School, Boston, MA 02115, United States; Department of Supportive Oncology, Dana-Farber Cancer Institute, Boston, MA 02215, United States; Brigham and Women’s Hospital, Boston, MA 02115, United States; Harvard Medical School, Boston, MA 02115, United States

**Keywords:** natural language processing, artificial intelligence, computing methodologies, electronic health records

## Abstract

**Objectives:**

Extracting sections from clinical notes is crucial for downstream analysis but is challenging due to variability in formatting and labor-intensive nature of manual sectioning. This study develops a pipeline for automated note sectioning using open-source large language models (LLMs), focusing on three sections: History of Present Illness, Interval History, and Assessment and Plan.

**Materials and Methods:**

We fine-tuned three open-source LLMs to extract sections using a curated dataset of 487 progress notes, comparing results relative to proprietary models (GPT-4o, GPT-4o mini). Internal and external validity were assessed via precision, recall, and F1 score.

**Results:**

Fine-tuned Llama 3.1 8B (F1 = 0.92) outperformed GPT-4o. On the external validity test set, performance remained high (F1 = 0.85).

**Discussion:**

While proprietary LLMs have shown promise, privacy concerns limit their utility in medicine; fine-tuned, open-source LLMs offer advantages in cost, performance, and accessibility.

**Conclusion:**

Fine-tuned, open-source LLMs can surpass proprietary models in clinical note sectioning.

## Background and significance

Clinical documentation is critical for patient care, facilitating communication across clinicians and providing a comprehensive record of patient progress from inpatient to outpatient settings. While clinical notes often follow semi-structured formats, such as SOAP or sectioned templates (eg, History of Present Illness, Family History, Review of Systems, Physical Exam, Assessment, and Plan), they also contain rich, unstructured free-text narratives documenting a clinician’s direct observations and assessments.^1^ Though unstructured/semi-structured free text contains valuable clinical information, the variability in formatting between individual documenting clinicians presents a challenge in the research setting. Manually “sectioning” of notes to find current information is labor-intensive, error-prone, and unsuitable for large-scale data analysis.^2^ Prior efforts to automate this process have included rule-based heuristics and machine learning models^3–5^; however, these approaches have limited generalizability across diverse note types, hospital systems, and clinical domains.

The emergence of large language models (LLMs) presents a transformative opportunity for section segmentation in clinical documentation.^6^ Unlike earlier approaches, LLMs are trained on diverse datasets, enhancing their adaptability to varied formats and institutions.^7^ Successful implementation of these methods could enable streamlined work- flows, focusing on extracting and analyzing specific sections of interest from clinical notes. A previous study found that proprietary LLMs, such as OpenAI’s GPT-4, achieved an average F1 score of 0.77 in identifying note sections.^6^ While this represents a promising initial result, access to these models is often limited due to privacy concerns. This study also tested open-source models but reached a lower performance than GPT-4. Our work implements a similar methodology, but focuses on specific sections of interest and a curated dataset to achieve state-of-the-art performance on this task with smaller fine-tuned LLMs (<8 billion parameters). We test for robustness using data from various cancer centers and institutions. By optimizing smaller models for targeted domains, such as History of Present Illness, Interval History, and Assessment and Plan, we aim to create accessible methods that improve efficiency in extracting sections of interest from clinical notes for downstream analysis.

## Objective

This study aims to develop an automated method to extract clinically relevant sections of notes essential for downstream analysis, using a scalable pipeline compatible with local and cloud hardware.

## Materials and methods

### Dataset

Clinical notes from three oncology groups (breast, gastrointestinal, neurological) were annotated by two nurse practitioners (KS and JB). The first 25 notes from the gastrointestinal group were independently coded to facilitate initial data familiarization and the development of a codebook. Using this preliminary codebook, KS and JB independently coded a total of 653 notes, identifying spans related to the history of present illness, interval history, and assessment & plan (A&P). Due to variability in documentation, the history of present illness and interval history were combined into a single label, recent clinical history (RCH).

Inter-rater reliability was calculated using Jaccard Index (JI).^7^ For sections where the JI between the two annotations exceeded 80%, the union of the annotations was adopted as the final label. A total of 125 notes did not meet this threshold and were re-coded through group discussion involving all annotators and a third-party adjudicator (JD). This process resulted in the finalized codebook (Appendix S1). An additional 494 notes were single coded by KS using the finalized codebook, culminating in a dataset of 1147 clinical notes (Table 1).

**Table 1.** Description of the dataset.

	All notes	Breast	GI	Neuro
No. of notes	1147	487	465	195
No. of unique patients	433	157	254	22
Provider (%)				
* Physician*	61.7	68.0	59.8	50.8
* Nurse practitioner*	29.7	25.3	29.0	42.6
* Physician assistant*	8.5	6.8	11.2	6.7
Average no. of tokens (95% CI)	1814 (1737-1891)	1789 (1671-1907)	1942 (1813-2071)	1570 (1737-1726)
Notes containing (%)				
* Recent clinical history*	86.6	86.0	92.5	73.8
* Assessment and plan*	87.2	87.3	92.5	74.4

### Baseline evaluation

For baseline evaluation, we tested two rule-based approaches: SecTag and the sectioner module from MedSpaCy.^3^^,^^4^ SecTag employs terminology-based rules and naive Bayesian scoring to identify section headers in clinical notes, while MedSpaCy, an updated version of SecTag used by the VA in multiple studies,^8^^,^^9^ builds upon this methodology. Both tools were adapted for compatibility with our processing pipeline.

In addition to these baselines, we utilized a Clinical-Longformer with a 4096-token context window,^10^ trained with a custom head to predict the start and end positions of target sequences. Using a dataset of 487 notes from the breast cancer center, we trained two separate models: one for extracting RCH and another for A&P.

### Models

Five LLMs [GPT-4o, GPT-4o mini,^11^ Llama 3.2 instruct (1B), Llama 3.2 instruct (3B), Llama 3.1 instruct (8B)^12^] were evaluated for section identification. OpenAI models ran on a HIPAA-compliant endpoint,^13^ while Meta models were run on a virtual machine with a context window of 8192 tokens. All used a unified prompt (Appendix S2); OpenAI models applied function-calling, and Meta models were tested pre and post supervised fine-tuning (SFT).^14^ Pre SFT inference was done with grammar to enforce output structure. Llama models were selected for SFT because of their accessibility and widespread adoption in clinical informatics research.^15^ All fine-tuning and inference was performed on a HIPAA-secure virtual machine equipped with an A100 40 GB GPU.

### Fine-tuning

We performed supervised fine-tuning of the LLMs using the Unsloth library.^16^ The models were trained using rank-stabilized LoRA,^17^ a parameter-efficient fine-tuning method that improves on the popular LoRA algorithm^18^ and showed better performance in our experiments. The training parameters were found through initial exploration: rsLoRA rank and alpha of 16, 5 epochs, batch size of 2 and learning rate of 2e-4. The fine-tuning dataset corresponded to the notes from the breast cancer center (*n* = 487), with no patient overlap with our test set. The fine-tuning process took one hour with the largest model (Llama 3.1 8B) and twenty minutes with the smallest model (Llama 3.2 1B).

### Postprocessing

**Figure 1.**
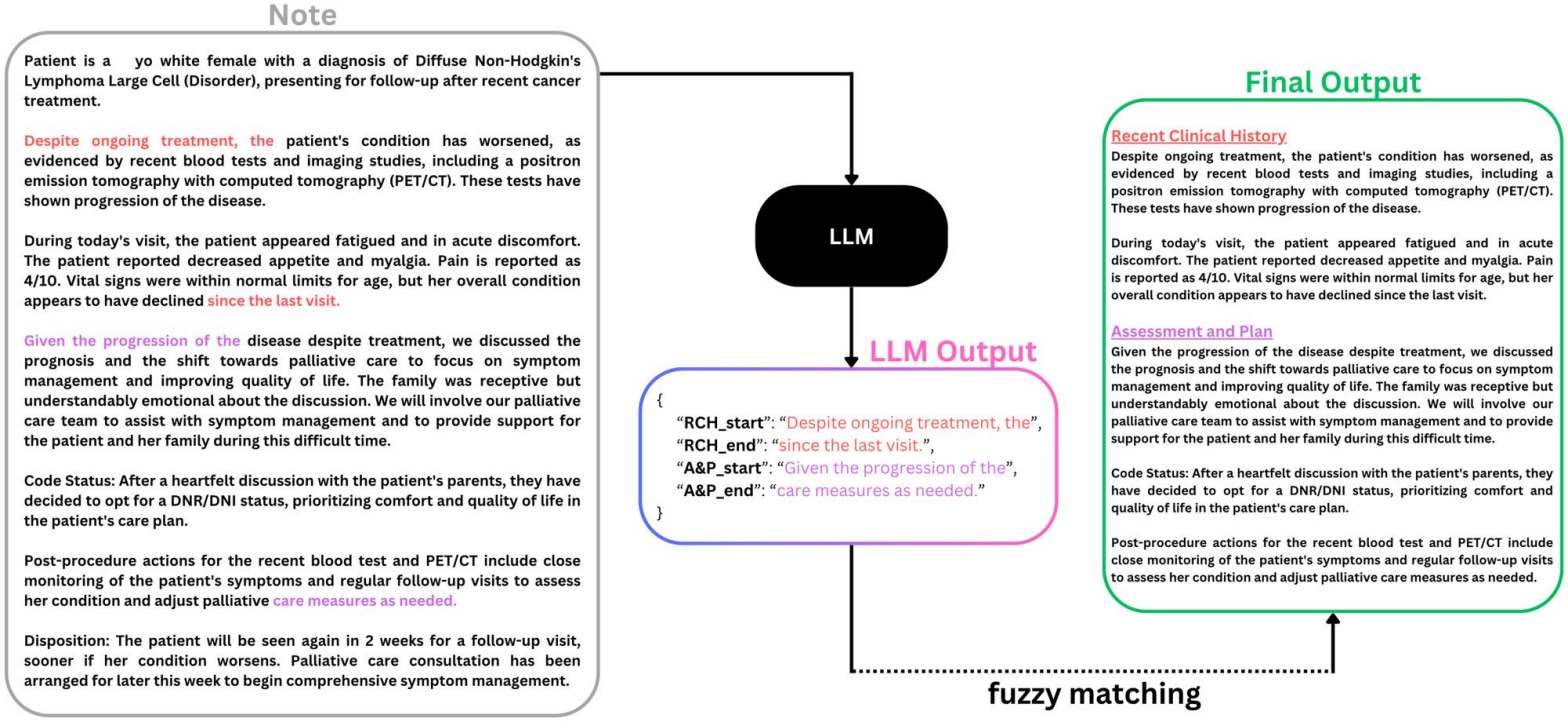
LLM workflow using fuzzy matching to extract clinical note sections.

An evaluation pipeline was implemented to process model outputs for each section of interest. Using vLLM^19^ to perform inference, the model was prompted to generate the first five words and the last five words of each predicted span.^6^ These 5-grams were compared to the source text to identify matches. If a match was found, the segment from the identified starting position to the identified ending position was extracted and labeled as the “predicted output” (Figure 1).

Due to the generative nature of LLMs, achieving an exact 5-gram match was uncommon, as observed in prior studies and in our experience.^6^ To address this, fuzzy matching was employed to align the predicted start and end strings with the source text. This process used a sliding window of 5-grams derived from the source text and assessed similarity using the Levenshtein distance,^20^ which measures the minimal number of edits required to transform one string into another. Matches with a similarity score exceeding 80% were considered valid, ensuring robust identification of spans in the generated output that closely align with the source text.

### Evaluation

**Figure 2.**
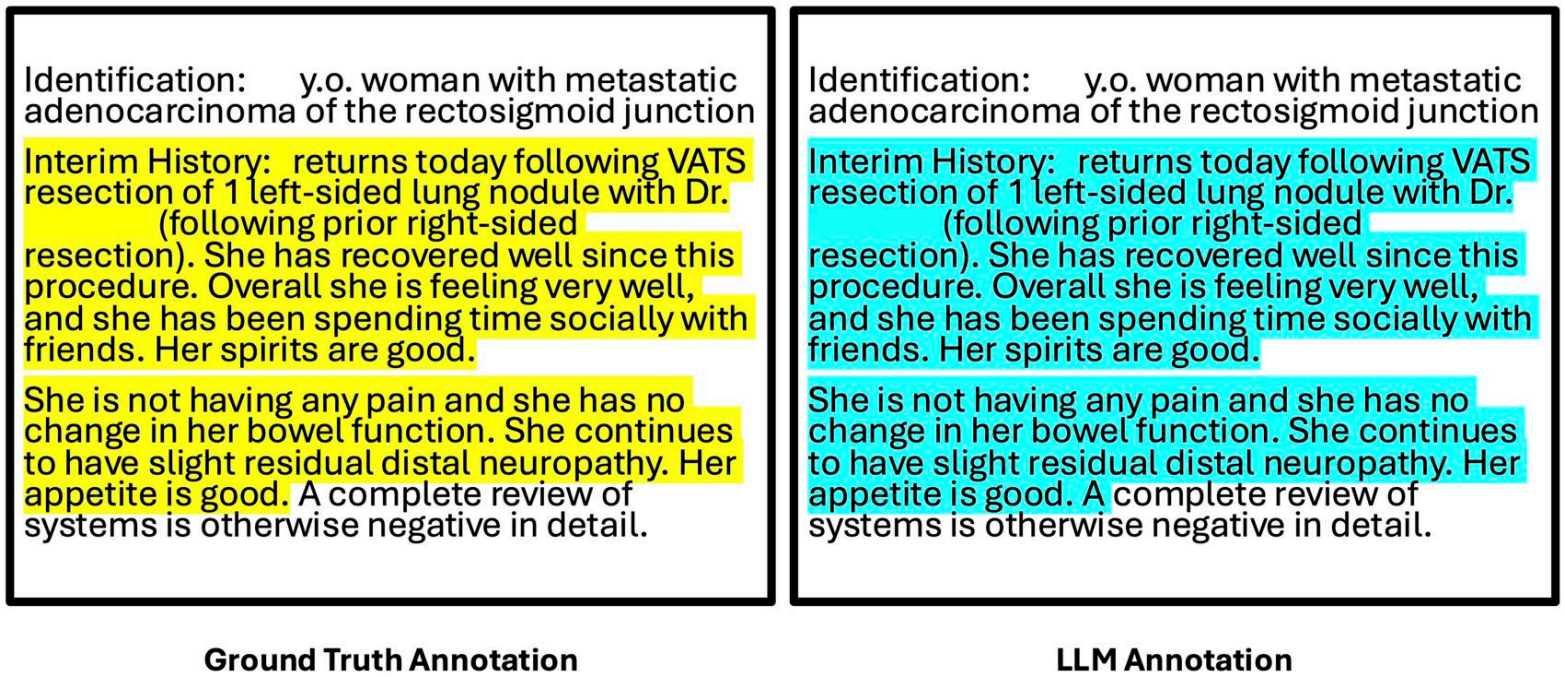
Comparison of ground truth and LLM-generated annotations for recent clinical history documentation.

The predicted outputs were compared to ground truth annotations (Figure 2), and precision, recall, and F1 score were calculated. To assess model performance, we first ran inference three times on each model, then bootstrapped (*n* = 1000) each run to obtain 3000 sets of metrics for evaluation. Statistical significance was assessed using a Friedman test (α = 0.05),^21^ with post-hoc pairwise comparisons via the Wilcoxon signed-rank test and a Bonferroni adjusted alpha of 0.01.^22^^,^^23^

### Internal validity

Outputs were evaluated on notes from two cancer centers (gastrointestinal and neurological), distinct from the cancer center used for training (breast), to assess performance across different patient populations at one institution.

### External validity

To evaluate the external validity of this method, the best-performing model was used to section 50 progress notes from breast cancer patients at UCSF.^24^ To ensure label consistency each note was annotated by KS using the validated codebook, and F1 scores were calculated.

## Results

### Baseline model performance

SecTag achieved an F1 score of 0.30 on the A&P section but was unable to generate a valid output for the RCH section. The average F1 scores across both labels for MedSpaCy and Clinical Longformer were 0.19 and 0.62, respectively. Detailed results of each approach can be found in Appendix S3.

### Performance of fine-tuned language models

We found that using SFT, the open source LLMs generated higher quality outputs relative to their base counterpart without the need for enforced structure (base model performance can be found in Appendix S4). Llama 3.1 8B had F1 scores of 0.89 and 0.94 for RCH and A&P respectively (Table 2). The difference in model performance was statistically significant (*P* < .01). Notably, Llama 3.1 8B scored 9-16 points higher than GPT-4o.

**Table 2.** Average performance of LLMs with 95% confidence intervals.

Model	GPT-4o mini		GPT-4o		Llama 3.2 1B cInstruct (FT)		Llama 3.2 3B Instruct (FT)		Llama 3.1 8B Instruct (FT)	
	RCH	A&P	RCH	A&P	RCH	A&P	RCH	A&P	RCH	A&P
F1 score (95% CI)	0.68 (0.65-0.71)	0.72 (0.69-0.74)	0.78 (0.75-0.81)	0.79 (0.77-0.82)	0.81 (0.78-0.83)	0.90 (0.88-0.92)	0.88 (0.86-0.90)	0.92 (0.90-0.93)	0.89 (0.87-0.91)	0.94 (0.93-0.95)
Precision (95% CI)	0.69 (0.66-0.73)	0.72 (0.69-0.75)	0.78 (0.75-0.81)	0.79 (0.76-0.81)	0.82 (0.79-0.85)	0.91 (0.89-0.93)	0.90 (0.88-0.92)	0.94 (0.92-0.95)	0.90 (0.89-0.92)	0.94 (0.93-0.96)
Recall (95% CI)	0.80 (0.77-0.82)	0.86 (0.84-0.88)	0.86 (0.83-0.88)	0.88 (0.86-0.90)	0.85 (0.83-0.88)	0.92 (0.90-0.94)	0.90 (0.89-0.92)	0.91 (0.90-0.93)	0.91 (0.90-0.93)	0.95 (0.94-0.96)

Error analysis was conducted on the top-performing model, Llama 3.1 8B, focusing on instances where the F1 score for a section fell below 0.8 (Gastrointestinal *n* = 96, Neurological *n* = 24). The most common error was over/under- prediction of target section; detailed error analysis can be found in Appendix S5.

### External validation

On the 50 external progress notes, the F1 scores for RCH and A&P using Llama 3.1 8B were 0.82 and 0.87 respectively.

## Discussion

This study demonstrates that small, fine-tuned language models can outperform proprietary models in clinical section segmentation, offering significant advantages in cost, accuracy, and accessibility. Unlike proprietary models requiring institutional agreements and high computational costs,^13^ our approach enables deployment on local or cloud-based systems, making it usable by researchers operating under resource constraints. This adaptability is crucial for downstream tasks such as symptom analysis and cohort discovery, where high-quality, actionable insights are critical.

Our findings demonstrate the potential of fine- tuning models with small datasets (fewer than 500 notes) to effectively perform note sectioning, even in the face of variability across clinical notes from different patient populations, offering a robust and adaptable solution for institutional use. Testing on notes from two distinct cancer populations and the progress notes of another institution highlights this approach’s internal and external validity. While our study focused on progress notes, the strong performance demonstrates that fine-tuned models may effectively adapt to variations in note structure and content across institutions.

By integrating note sectioning with a small language model as a preprocessing step, the input size for larger, more resource-intensive language models in downstream tasks is significantly reduced. This reduction in input size decreases computational demands, leading to lower energy consumption and, consequently, a reduced carbon footprint.^25^ This approach underscores the potential for sustainable AI practices in clinical data processing by optimizing resource usage without compromising performance.

By providing a cost-effective and privacy-conscious solution, this work reduces reliance on proprietary systems. The affordability and accessibility of our approach ensures that high-quality research is no longer limited to large institutions, fostering innovation across diverse settings.

### Limitations

While the model demonstrated strong performance overall, error analysis revealed patterns of overprediction and underprediction, particularly in sections with ambiguous or inconsistent boundaries. These errors highlight challenges posed by variability in clinical note structures and suggest areas for improvement, such as incorporating additional section labels to enhance discriminatory power. A potential mitigation strategy is incorporating a human-in-the-loop step to ensure sectioning aligns with study standards.^26^

This study focused exclusively on notes authored by physicians, nurse practitioners, and physician assistants, without evaluating notes written by other clinical staff, such as physical therapists, occupational therapists, or nutritionists. Furthermore, all analyzed notes originated from academic medical centers, limiting the assessment of variability in note styles across different types of hospital systems, such as community hospitals.

## Conclusion

Our method demonstrates a robust, institution-agnostic solution for segmentation of clinical notes. By leveraging fine-tuned models that are cost-effective and adaptable, this approach offers a scalable and accessible methodology for improving clinical documentation analysis across diverse healthcare settings.

## Supplementary Material

ooaf179_Supplementary_Data

## Data Availability

The code used for this project as well as sample annotations based on the CORAL dataset are available in the following repository: https://github.com/lindvalllab/MedSlice.
