# Supplementary material for: MedSlice: fine-tuned large language models for secure clinical note sectioning
Source: JAMIA Open. 2026 Jan 13;9(1):ooaf179. doi: 10.1093/jamiaopen/ooaf179 (PMC12803779; doi:10.1093/jamiaopen/ooaf179)
Supplement: ooaf179_Supplementary_Data [file ooaf179_supplementary_data.docx]

**Appendix 1: Annotation Codebook**

***in general stick with annotating in big chunks rather than separated sections***

- Not possible if HPI and interval history are separated by a large chunk of the oncology history and that’s ok

**Recent Clinical History:**

Include:

- Anything in the following section heads/content:
  - Interval History, interval treatment
  - Subjective
  - HPI, even if there is a lot of past Onc info In it, unless there is a separate section labeled Onc hx then can omit that
- Free-hand documentation (e.g. unstructured communication notes with a patient at the bedside/clinic) that appear to be written without template.
- Text which looks like past interval history or past HPI but is not clearly demarcated by either a title (“HPI”) phrases, or another indication.
- Talk of a list of current symptoms that is outside the standard ROS and can clearly be seen as free text documentation from the encounter
  - “no nausea or itching”

Exclude:

- The following sections: (even if they have something that might look important as it will be discussed again later on)
  - Chief complaint
  - Patient ID
  - Reason for visit – UNLESS the words in there are the HPI!!
  - Oncology history
  - Review of systems
  - Current treatment/therapy
  - Templated lists of ESYM responses
  - Patient instructions
  - Referral orders
- Information that is clearly copied forward, typically starts with or is followed by one of these sentences: Copied from, Above is for reference only, For reference, Carried through for continuity, Above history is for clinical reference only, Oncology history overview, OncHx has been copied forward and edited/updated from prior documentation for the purpose of clinical reference only, Oncology History, PMH, FH, and SH copied forward from previous notes and updated, included for clinical reference only.

**Assessment and Plan:**

Include:

- Beginning at assessment and ending at the end of the follow up instructions
- Attending attestations (Just continue the same block of labeled text even if you include some things you normally would not)
- Statements about follow up timing if it seems to be free text or there are clinical implications or information presents
- “IMP” = impression
- “Impression and recommendations”

Exclude:

- Information that is copied forward: “Last assessment and plan”
- Billing statements
- “Verbalized understanding, all questions answered, will call…” unless it has non templated writing like “for worsening pain”
- Attestations if there is no free written text, and is just templated language, i.e.; “I agree with assessment and plan with PA above”

**Appendix 2: Prompt used for all LLMs**

“Your task is to find the parts of a clinical note corresponding to the sections -History of Present Illness and Interval History-, and -Assessment and Plan-. You should organize this information in a JSON output that extracts the first and last five words for each of these sections. If the sections HPI_Interval_Hx or A&P are not in the medical note, return an empty string for the corresponding section's start and end. Below is the medical note:”

**Appendix 3: Evaluation of sectioning approaches found in the literature**

| **Model** | **SecTag** | | **MedSpaCy** | | **Clinical-Longformer** | |
| --- | --- | --- | --- | --- | --- | --- |
|  | **RCH** | **A&P** | **RCH** | **A&P** | **RCH** | **A&P** |
| **F1 Score** | - | 0.3 | 0.21 | 0.16 | 0.81 | 0.63 |
| **Precision** | - | 0.31 | 0.24 | 0.16 | 0.82 | 0.64 |
| **Recall** | - | 0.42 | 0.21 | 0.16 | 0.84 | 0.65 |

**Appendix Table 4: Performance of base models**

| **Model** | **Llama 3.2 1B Base** | | **Llama 3.2 3B Base** | | **Llama 3.1 8B Base** | |
| --- | --- | --- | --- | --- | --- | --- |
|  | **RCH** | **A&P** | **RCH** | **A&P** | **RCH** | **A&P** |
| **F1 Score**  **(95% CI)** | 0.14  (0.12–0.16) | 0.11  (0.09–0.13) | 0.35  (0.32–0.38) | 0.45  (0.42–0.48) | 0.53  (0.50–0.55) | 0.51  (0.48–0.54) |
| **Precision**  **(95% CI)** | 0.21  (0.18–0.24) | 0.12  (0.09–0.14) | 0.52  (0.49–0.56) | 0.55  (0.52–0.59) | 0.69  (0.66–0.73) | 0.54  (0.50–0.57) |
| **Recall**  **(95% CI)** | 0.14  (0.11–0.16) | 0.11  (0.09–0.13) | 0.40  (0.37–0.43) | 0.54  (0.51–0.57) | 0.51  (0.48–0.53) | 0.68  (0.65–0.70) |

**Appendix Table 5: Error Analysis of Llama 3.1 8B Inference on Gastrointestinal and Neurological Notes**


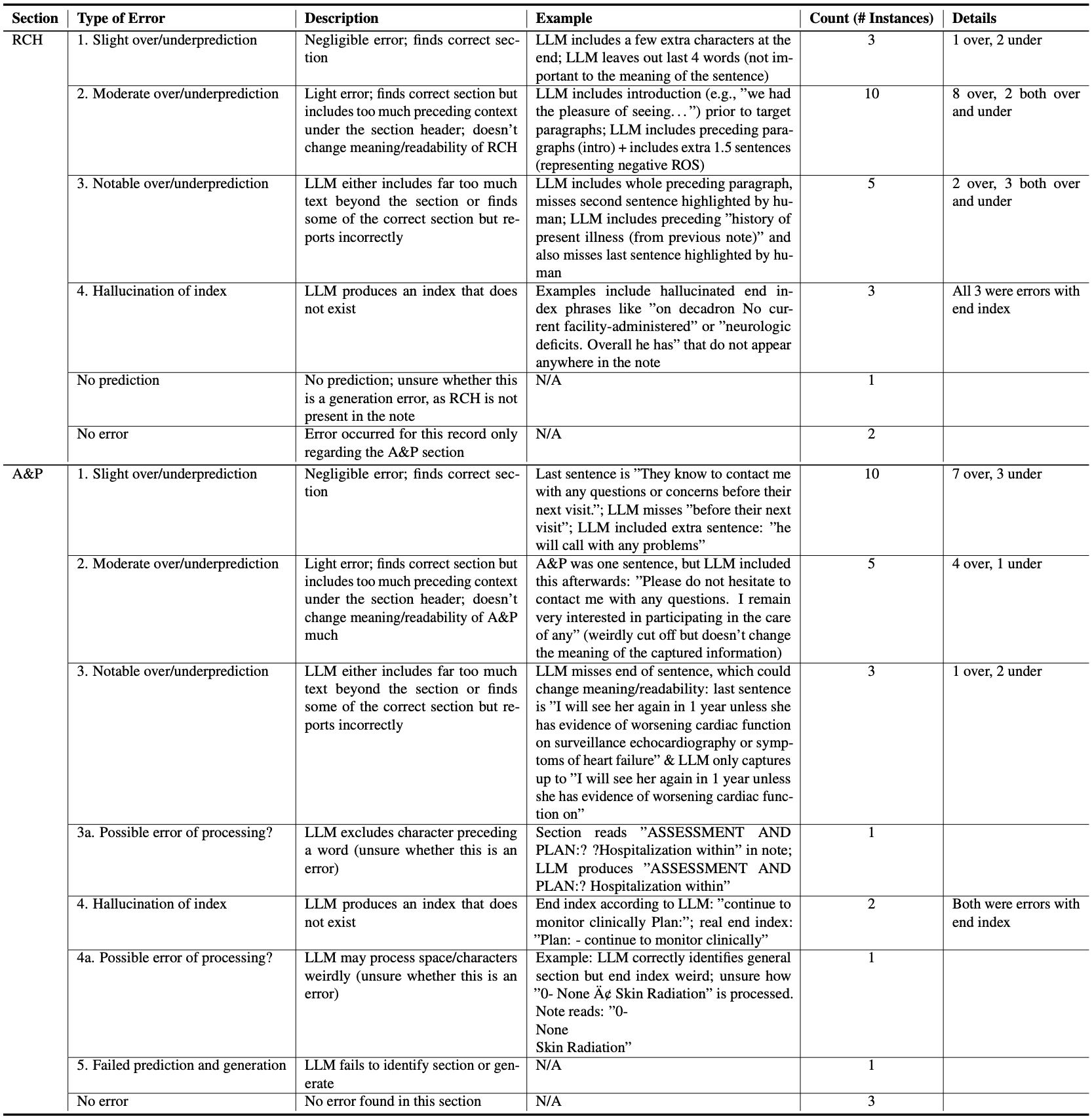


Neurological


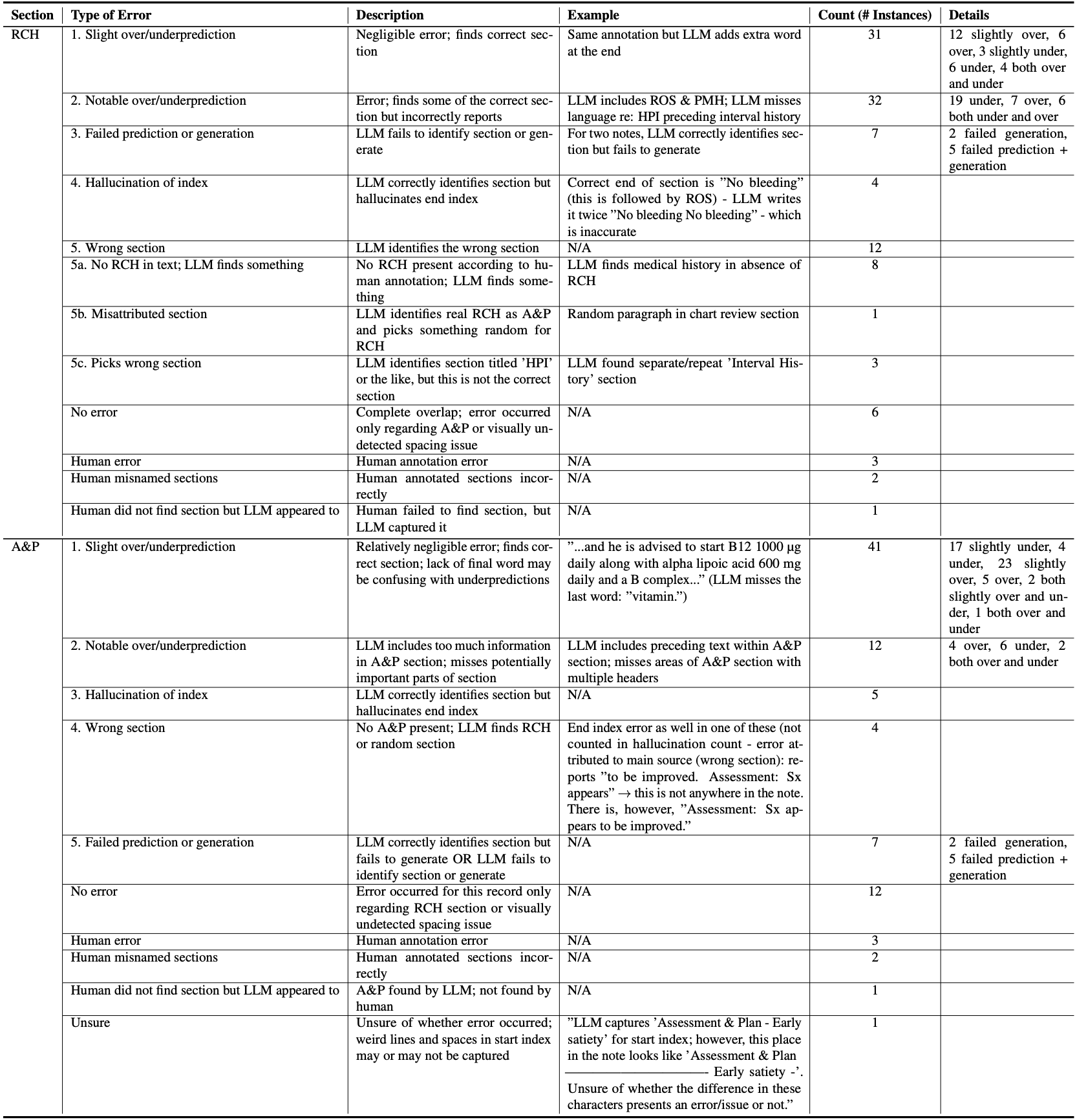


Gastrointestinal
